# Binary Matrix Shuffling Filter for Feature Selection in Neuronal Morphology Classification

**DOI:** 10.1155/2015/626975

**Published:** 2015-03-29

**Authors:** Congwei Sun, Zhijun Dai, Hongyan Zhang, Lanzhi Li, Zheming Yuan

**Affiliations:** ^1^Hunan Provincial Key Laboratory of Crop Germplasm Innovation and Utilization, Hunan Agricultural University, Changsha, Hunan 410128, China; ^2^Hunan Provincial Key Laboratory for Biology and Control of Plant Diseases and Insect Pests, Hunan Agricultural University, Changsha, Hunan 410128, China; ^3^College of Information Science and Technology, Hunan Agricultural University, Changsha, Hunan 410128, China

## Abstract

A prerequisite to understand neuronal function and characteristic is to classify neuron correctly. The existing classification techniques are usually based on structural characteristic and employ principal component analysis to reduce feature dimension. In this work, we dedicate to classify neurons based on neuronal morphology. A new feature selection method named binary matrix shuffling filter was used in neuronal morphology classification. This method, coupled with support vector machine for implementation, usually selects a small amount of features for easy interpretation. The reserved features are used to build classification models with support vector classification and another two commonly used classifiers. Compared with referred feature selection methods, the binary matrix shuffling filter showed optimal performance and exhibited broad generalization ability in five random replications of neuron datasets. Besides, the binary matrix shuffling filter was able to distinguish each neuron type from other types correctly; for each neuron type, private features were also obtained.

## 1. Introduction

To accelerate the understanding of neuronal characteristics in the brain, the prerequisite is to classify neurons correctly. It is therefore necessary to develop a uniform methodology for their classification. The existing classification techniques are usually based on structural functions and the numbers of dendrites to fit the models [1]. As neuronal morphology is closely related to neuronal characteristics and functions, neuroscientists have been making great efforts to study neurons from the perspective of neuronal morphology. Renehan et al. [2] employed intracellular recording and labeling techniques to examine potential relationships between the physiology and morphology of brainstem gustatory neurons and demonstrated a positive correlation between the breadth of responsiveness and the number of dendritic branch points. In the study by Badea and Nathans [3], detailed morphologies for all major classes of retinal neurons in adult mouse were visualized. After analyzing the multidimensional parametric space, the neurons were clustered into subgroups by using Ward's and *K*-means algorithms. In the study by Kong et al. [4], retinal ganglion cells were imaged in three dimensions and the morphologies of a series of 219 cells were analyzed. A total of 26 parameters were studied, of which three parameters, level of stratification, extent of the dendritic field, and density of branching, were used to get an effective clustering, and the neurons could often be matched to ganglion cell types defined by previous studies. In addition, a quantitative analysis based on topology and seven morphometric parameters was performed by Ristanović et al. in adult dentate nucleus [5], and neurons were classified into four types in this region. A number of neuronal morphologic indices such as soma surface, number of stems, length, and diameter were designed [6], which makes it possible to classify neurons based on morphological characteristics.

In the study by Li et al. [7], a total of 60 neurons were selected randomly and five of the twenty morphologic characteristics were extracted by principal component analysis (PCA), after which neurons were clustered into four types. Jiang et al. [8] extracted four principal components of neuronal morphology by PCA and employed back propagation neural network (BPNN) to distinguish the same kinds of neuron in different species. However, the above studies [2–5] only focused on a particular neuronal type or specific region of the brain, aiming to solve specific issues rather than classify neurons systematically. In this form, only a few samples were selected and the classification results were not independently tested, which is not persuasive enough. Moreover, the methodologies used in previous studies [7, 8] were mainly based on PCA and cluster analysis. PCA is the optimal linear transformation to minimize the mean square reconstruction error, but it only considers second order statistics, and if the data have nonlinear dependencies, higher order statistics should be taken into account [9]. Besides, the principal component was a compression of attributes, and it was hard to interpret the respective contribution. Therefore, feature selection (FS) is necessary, which is able to simplify the model by removing redundant and irrelevant features.

Available feature selection methods fall into three categories, (i) filter methods, in which inherent features of datasets are used to rank variables, and the algorithm complexities are low. However, redundant phenomena are usually present among the selected features, which may result in low classification accuracy. Univariate filter methods include *t*-test [10], correlation coefficient [11], Chi-square statistics [12], information gain [13], relief [14], signal-to-noise ratio [15], Wilcoxon rank sum [16], and entropy [17]. Multivariable filter methods include mRMR [18], correlation-based feature selection [19], and Markov blanket filter [20]. There are also (ii) wrapper methods, where the training precision and algorithm complexity are high, which usually leads to overfitting. Representative methods include sequential forward selection [21], sequential backward selection [21], sequential floating selection [22], genetic algorithm [23], and ant colony algorithm [24]. SVM and ANN are usually used for implementation. There are also (iii) embedded methods, including support vector machine recursive feature elimination (SVM-RFE) [25], support vector machine with RBF kernel based on recursive feature elimination (SVM-RBF-RFE) [26], support vector machine and T statistics recursive feature elimination (SVM-T-RFE) [27], and random forest [28], which use internal information of the classification model to evaluate selected features.

In this work, a new feature selection method named BMSF was used. It not only overcame over fitting problem in a large dimensional search space but also took potential feature interactions into account during feature selection. Seven types of neurons, including pyramidal neuron, Purkinje neuron, sensory neuron, motoneuron, bipolar interneuron, tripolar interneuron, and multipolar interneuron, that have different characteristics and functions in the NeuroMorpho.org database were selected, being derived from all the existing species or brain regions (up to version 6.0). BMSF was used to reduce features nonlinearly, and support vector classification (SVC) model was built to classify neurons based on the reserved morphological characteristics. SVM-RFE and rough set theory were used to give a comparison with the introduced feature selection methods, while another two classifiers including the back propagation neural network (BPNN) and Naïve Bayes (NB), which are widely used in the pattern recognition field, were employed to test the robustness of the BMSF. A systematic classification of neurons would facilitate the understanding of neuronal structure and function.

## 2. Materials and Methods

### 2.1. Data Sources

Data sets used in this work were downloaded from the NeuroMorpho.org database [6, 29]. NeuroMorpho.org is a web-based inventory dedicated to densely archiving and organizing all publicly shared digital reconstructions of neuronal morphology. NeuroMorpho.org was started and maintained by the Computational Neuroanatomy Group at the Krasnow Institute for Advanced Study, George Mason University. This project is part of a consortium for the creation of a “neuroscience information framework,” endorsed by the Society for Neuroscience, funded by the National Institutes of Health, led by Cornell University (Dr. Daniel Gardner), and including numerous academic institutions such as Yale University, Stanford University, and University of California, San Diego (http://neuromorpho.org/neuroMorpho/myfaq.jsp). The data sets used in this study were documented in Table 1. A total of 5862 neurons were selected, and training and test sets were divided randomly in the percentage of 2 : 1 in each neuron type. Finally, we obtained five pairs of data sets, each with random samples.

### 2.2. Feature Extraction and Selection

Dendritic cells in the NeuroMorpho.org database were cut into a series of compartments, and each compartment was characterized by an identification number, a type, and the spatial coordinates of the cylinder ending point, the radius value, and the identification number of the “parent.” Although the digital description constituted a completely accurate mapping of dendritic morphology, it bore little intuitive information [30]. In this work, 43 attributes that held more intuitive information were extracted with L-measure software [31], and related morphological indices and descriptions are shown in Table 2. For convenience, we gave an abbreviation for each neuronal morphological index, as listed in the second column of Table 2.

It was considered redundant among attributes. Feature selection was able to save the cost of computational time and storage and simplify models when dealing with high dimensional data sets, and it was also useful to improve classification accuracy by removing redundant and irrelevant features.

#### 2.2.1. Binary Matrix Shuffling Filter

For rapid and efficient selection of high-dimensional features, we have reported a novel method named binary matrix shuffling filter (BMSF) based on support vector classification (SVC). The method was successfully applied to the classification of nine cancer datasets and obtained excellent results [32]. The outline of the algorithm is as follows.

Firstly, denoting the original training set as (*y*_*i*_, *x*_*i*,*j*_), which includes *n* samples and *m* features, where *i* = 1,2,…, *n*, *j* = 1,2,…, *m*, we randomly generate a matrix with dimensions *k* × *m* with entries being either 1 or 0, representing whether the feature in that column is included in the modeling or not. Where *k* is the given number of combinations (*k* = 50 in this paper), the number of 1 or 0 in each column (each feature) is equal.

Secondly, for each combination, there will be a reduced training set from the original training set according to the subscripts of those selected features, and classification accuracy can be obtained through tenfold cross validation. By repeating this process *k* times, *k* values of accuracy are obtained.

Thirdly, taking the *k* values of accuracy as the new dependent variable, the *k* × *m* random 0 or 1 matrix as the independent variable matrix, a new training set is constructed. To evaluate the contribution of a single feature to the model, we change all the 1 in *j*th column to 0 and all the 0 in that column to 1 (keeping the other columns unchanged) to produce two test sets with all the elements of 0 or 1 in *j*th column. The newly produced training set is used to build the model to predict the two kinds of test sets, and the predictive vectors *Z*_1_ and *Z*_0_ are then obtained.

Comparing the mean value of vectors *Z*_1_ and *Z*_0_, if the mean value of *Z*_1_ is bigger than that of *Z*_0_, the feature corresponding to this column tends to give better classification performance. Otherwise, this feature should be excluded. Repeating this process, the features are screened in multiple rounds until no more can be deleted.

Detailed procedures can be found in our previous study [32]. This method is able to find a parsimonious set of features which has high joint prediction power.

#### 2.2.2. Support Vector Machine Recursive Feature Elimination

SVM-RFE is an application of recursive feature elimination (RFE) using the weight magnitude as the ranking criterion. It eliminates redundant features and yields more compact feature subsets. The features are eliminated according to a criterion related to their support to the discrimination function, and the support vector is retrained at each step. This method was first successfully used in gene feature selection and afterwards in the fields of bioinformatics, genomics, transcriptomics, and proteomics. For the technical details of the method, refer to the original study by Guyon et al. [25].

#### 2.2.3. Rough Set Theory

Rough set theory, introduced by Pawlak [33] in the early 1980s, is a tool for representing and reasoning about imprecise and uncertain data. It constitutes a mathematical framework for inducing minimal decision rules from training examples. Each rule induced from the decision table identifies a minimal set of features discriminating one particular example from other classes. The set of rules induced from all the training examples constitutes a classificatory model capable of classifying new objects. The selected feature subset not only retains the representational power but also has minimal redundancy. A typical application of the rough set method usually includes three steps: construction of decision tables, model induction, and model evaluation [34]. The algorithm used in this work is derived from the study by Hu et al. [35–37].

### 2.3. Classification Techniques

#### 2.3.1. Support Vector Classification

Support vector classification, based on statistic learning theory, is widely used in the machine learning field [38]. In SVM, structural risk minimization is a substitution of traditional empirical risk minimization, and it is particularly suitable for small sample size, high-dimensional, nonlinearity, overfitting, dimension disaster, local minima, and strong collinear problems. Meanwhile, it also performs excellent generalization abilities. In this work the nonlinear radial basis function (RBF) was selected, where the ranges of parameters *c* and *g* for optimization were −5 to 15 and 3 to −15 (base-2 logarithm), respectively. The cross validation and independent test were carried out using in-home programs written in MATLAB (version R2012a).

#### 2.3.2. Back Propagation Neural Network

BPNN is one of the most widely employed techniques among the artificial neural network (ANN) models. The general structure of the network consists of an input layer, a variable number of hidden layers containing any number of nodes, and an output layer. The back propagation learning algorithm modifies the feed-forward connections between the input and hidden units and the hidden and outputs units to adjust appropriate connection weights to minimize the error [39]. Java-based software WEKA [40] was used to fit the model.

#### 2.3.3. Naïve Bayes

Naïve Bayes is a classification technique obtained by applying a relatively simple method to a training dataset [41]. A Naïve Bayes classifier calculates the probability that a given instance belongs to a certain class. Considering its simple structure and ease of implementation, Naïve Bayes often performs well. Naïve Bayes models were also implemented in the WEKA software, and all the parameters were set by default.

## 3. Results and Discussion

### 3.1. Selected Feature Subsets

Feature selection methods are applied to training sets to get optimal feature subsets. For each method, five sets of features were obtained.  Table 3 shows the reserved feature subsets derived from BMSF, SVM-RFE, and rough set theory, respectively. Five feature subsets are numbered with Roman numerals I to V for five replications. The number of selected features is also listed in Table 3.

As shown in Table 3, approximately eight features on average were reserved by BMSF, while the number of features derived from SVM-RFE and rough set theory was more than ten. BMSF retained fewer features, which were more informative and easy to interpret. The feature ranking list showed the importance of a certain feature. In the feature subsets of BMSF and rough set, *N*_*s*_ ranked first in five replications, which indicated that *N*_*s*_ had a strong ability to discriminate neuron types. We calculated the frequency of each of the selected features in the five replications. Except for *N*_*s*_, features NW, HT, and Ta2 were also reserved in five random replications simultaneously, and their ranking lists were similar in the five BMSF subsets.

### 3.2. Classification Performance

#### 3.2.1. Comparison of Independent Test Accuracies Using Different Models

In order to evaluate the performance of BMSF and make a comparison with SVM-RFE and rough set, three classifiers were employed to perform independent test. Including the classification performance without features selection, there were twelve classification accuracies. The average accuracies on five random datasets are presented in Table 4.

The independent classification accuracy is the ratio of the total correctly classified samples to the total test samples. As shown in Table 4, of the twelve results obtained, the optimal classification model based on the five datasets is BMSF-SVC (97.84%), followed by SVC without feature selection (97.1%). Excellent classification results on the SVC classifier indicated that all the extracted features were useful in identifying neurons, and few irrelevant features were extracted. Further, after feature selection by BMSF, the classification accuracy of SVC increased. This phenomenon suggested that BMSF deleted redundant features successfully and simplified models with fewer features. On the other hand, the feature subsets derived from SVM-RFE and rough set did not contribute to increasing the accuracies on SVC; in fact, they decreased sharply. A similar finding can be found for Naïve Bayes, as the two feature selection methods decreased the performance of Naïve Bayes, while BMSF improved the performance. The classifier BPNN showed little sensitivity to feature subsets, and the classification performance was at similar levels. With fewer features, BMSF also obtained good accuracy on BPNN, and a simplified model may be useful in further interpretation.

The above independent accuracies indicated that BMSF has an excellent generalization ability and robustness on the three classifiers. We also calculated the average performance of each feature selection method on the three classifiers and the classification performance based on the three different feature selection methods. The results are listed in the last row and column of Table 4. The average classification accuracy based on BMSF was also the best.

As the datasets used in this work are unbalanced (as shown in Table 1), it is necessary to break down the independent test accuracy to obtain the classification performance of each cell type. Based on the predicted labels, the sensitivities of each cell type in the five replications are presented in Table 5.

For seven neuron types, BMSF-SVC exhibited the best performance on pyramidal neuron, motoneuron, sensory neuron, and bipolar neuron. Though tripolar and multipolar neurons showed excellent performance on Naïve Bayes, they did not do very well on other neuron types. The classification result of multipolar neuron was poor; however, SVM-RFE and rough set also performed less well on SVC. We found that the predicted labels of multipolar neuron are almost the same as those of the pyramidal neuron in all the models, which indicated that the unbalanced datasets had an effect on the prediction of multipolar neuron.

#### 3.2.2. Distinguishing a Certain Neuron Type from Others by BMSF-SVC

To evaluate whether a certain feature subset is useful in identifying only a single cell type, the optimal model (BMSF-SVC) in this study was employed. For seven neurons types, six hierarchy models were established. In each hierarchy model, it was a binary classification problem. Due to the imbalanced datasets in this paper, accuracy and the Matthews correlation coefficient (MCC) were used to evaluate the established models, and recall was used to evaluate the classification performance of single neuron type as follows:

(1) Accuracy=TP+TNTP+TN+FP+FN;MCC=TP×TN−FP×FNTP+FPTP+FNTN+FPTN+FN;Recall=TPTP+FN,

where TP, TN, FP, and FN were true positive, true negative, false positive, and false negative, respectively, which derived from the confusion matrix. In this paper, positive samples were a certain neuron type and all the rest of the neuron types were negative samples. Positive samples were selected according to the number of samples in each type, and the datasets in each hierarchy are presented in Table 6. For each neuron type, private feature subsets were obtained.

As shown in Table 6, the accuracies and MCC in each hierarchy indicated the effectiveness of the models. We obtained private feature subsets for each neuron type. These features were useful in identifying the corresponding neurons, and the perfect recall may support our conclusion. The above finding suggested that BMSF was not only useful in identifying all seven cell types but also able to discriminate specific neuron types.

In this paper, we used a new feature selection method named BMSF for neuronal morphology classification. Interactions are taken into consideration to get highly accurate classification of neurons, and this method usually selects a small amount of features for easy interpretation. As shown in Table 3, eight features were reserved via BMSF, which was less than the number of features obtained by the other two feature selection methods. The BMSF method automatically conducts multiple rounds of filtering and guided random search in the large feature subset space and reports the final list of features. Though this process is wrapped with SVC, the features selected have general applicability to multiple classification algorithms. This conclusion can be demonstrated by the classification performance shown in Table 4.

We should point out that different runs of BMSF may produce different lists of feature subsets. This phenomenon arises from the fact that there are many possible characteristics that may be used to distinguish neurons. For example, feature subsets derived from rough set theory and BMSF achieve similar classification accuracy when applied to SVC classifier. Our goal is to find a minimal set of such features that the combination of them can well differentiate the dependent variables.

The reserved feature subsets on the same data set that resulted from different feature selection methods differed greatly. Li et al. [7] and Jiang et al. [8] selected features from the first twenty attributes of Table 1 only, so they inevitably ignore the attributes that were reserved by BMSF. Therefore, feature extraction by L-measure software was necessary. Another drawback of their feature selection methods was that they did not reduce the variables in the nonlinear manner. For example, PCA only considers second order statistics, and interactions cannot be taken into account.

Conventional classification techniques were built on the premise that the input data sets were balanced; if not, the classification performance would decrease sharply [42]. There were 3908 neurons in the training set, but the number of neurons in each type differed greatly (Table 1). For example, there were only 24 and 11 multipolar interneurons and Purkinje neurons, respectively, whereas the number of pyramidal neurons was 3172, and the unbalanced data sets would have a negative effect on the classification results (Table 5). Therefore, we conducted the hierarchy model for each neuron type, and BMSF was demonstrated as useful in distinguishing specific neuron types from others.

## 4. Conclusion

We introduced a new feature selection method named BMSF for neuronal morphology classification, obtained satisfactory accuracy for all of the datasets and each hierarchy model, and were able to select private parsimonious feature subsets for each neuron type. However, it was obvious that classification based simply on neuronal morphology was inadequate. As time goes by, dendrites may continue to grow and axons will generate additional terminals, which will undoubtedly lead to changes in the vital parameters [8]. Therefore, combining biophysical characteristics with function characteristics to investigate the neuronal classification problem will be a productive direction in the future.

## Figures and Tables

**Table 1:** Summary of training and test sets numbers in each neuronal type.

	Neuron type	Number of training sets	Number of test sets	Total
1	Pyramidal	3172	1586	4758
2	Motoneuron	298	149	447
3	Sensory	261	130	391
4	Tripolar	94	48	142
5	Bipolar	48	24	72
6	Multipolar	24	12	36
7	Purkinje	11	5	16
Total		**3908**	**1954**	**5862**

**Table 2:** The 43 morphological characteristics extracted by L-measure software and their descriptions.

Number	Abbr.	Morphological index	Description
1	SS	Soma_surface	Somatic surface area
2	*N*_*s*_	N_stems	Total number of trees
3	*N*_bi_	N_bifs	Total number of bifurcations
4	*N*_br_	N_branch	Number of bifurcations plus terminations
5	*N*_*t*_	N_tips	Number of terminal tips of a neuron
6	NW	Neuronal_width	95% of second principal component
7	NH	Neuronal_height	95% of first principal component
8	ND	Neuronal_depth	95% of third principal component
9	Ty	Type	Compartments are assigned to four different types: 1 = soma, 2 = axon, 3 = dendrites, and 4 = apical dendrites
10	Di	Diameter	Average branch diameter
11	Dp	Diameter_pow	Diameter of each compartment of the neuron raised to the power of 1.5
12	Le	Length	Total arborization length
13	Su	Surface	Surface area of each compartment
14	SA	Section area	Total arborization surface area
15	Vo	Volume	Total internal volume of the arborization
16	ED	Euc distance	Maximum euclidean (straight) distance from soma to tips
17	PD	Path distance	Maximum path (along the tree) distance from soma to tips
18	BO	Branch_order	Maximum branch order number of bifurcations from soma to tips
19	Td	Terminal degree	Total number of tips each segment will terminate into
20	TS	Terminal segment	Number of compartments that comprise the terminal branch
21	Ta1	Taper_1	The change in diameter over path length between two critical points
22	Ta2	Taper_2	The ratio of the change in diameter to the initial diameter of two critical points. The initial diameter is usually larger
23	Bpl	Branch_path length	Summation of the individual compartment lengths that form a branch
24	Co	Contraction	Average contraction (the ratio between euclidean and path length calculated on each branch)
25	Fr	Fragmentation	Total number of reconstruction points
26	DR	Daughter_ratio	Ratio between the diameter of the bigger daughter and the smaller daughter of the current bifurcation
27	PDR	Parent-daughter_ratio	Ratio between the diameter of a daughter and its father for each critical point
28	Pa	Partition_asymmetry	Average over all bifurcations of the absolute value of (*n*1 − *n*2)/(*n*1 + *n*2 − 2), where *n*1 and *n*2 are the numbers of tips in the two subtrees
29	RP	Rall_power	Average over all bifurcations of the sum of the diameters of the two daughters, elevated to 1.5, divided by the diameter of the parent, and elevated to 1.5
30	Pk	Pk	*n* = [0, 5]
31	Pc	Pk_classic	Rall power is set to 1.5
32	Pk2	Pk_2	Rall power is set to 2
33	Bal	Bif_ampl_local	Average over all bifurcations of the angle between the first two daughter compartments
34	Bar	Bif_ampl_remote	Average over all bifurcations of the angle between the following bifurcations or tips
35	Btl	Bif_tilt_local	The angles between the end of the parent branch and the initial part of the daughter branches at the bifurcation
36	Btr	Bif_tilt_remote	The angles between the previous node of the current bifurcating father and the daughter nodes
37	Btol	Bif_torque_local	Angle between the current plane of bifurcation and the previous plane of bifurcation
38	Btor	Bif_torque_remote	Angle between the current plane of bifurcation and the previous plane of bifurcation
39	Lpd	Last_parent_diam	Diameter of last bifurcation before the terminal tips
40	Dt	Diam_threshold	Diameter of first compartment after the terminal bifurcation leading to a terminal tip
41	HT	Hillman threshold	Computation of the weighted average diameter between 50% of father and 25% of daughter diameters of the terminal bifurcation
42	He	Helix	Helicity of the branches of the neuronal tree. It needs to be at least 3 compartments long to compute the helicity
43	FD	Fractal_dim	Fractal dimension metric of the branches in the dendrite trees

**Table 3:** Summary of selected features.

Feature selection method	Feature subset	Number of features	Selected features
SVM-RFE	I	10	SS, HT, DR, Bpl, NH, Btr, Bal, Su, SA, Lpd
	II	13	HT, RP, SS, Ta1, Btr, BO, Dp, Di, Td, Fr, DR, Bar, NH
	III	12	HT, FD, SS, DR, Btr, Dp, Di, Fr, BO, Td, Su, Ty
	IV	14	HT, RP, SS, Ta2, Btr, Di, Dp, Fr, BO, Td, SA, Vo, Ta1, TS
	V	15	HT, Lpd, SS, Bpl, Btr, Bal, NH, Ta1, Su, Di, SA, Vo, Fr, Ta2, Ty

Rough set	I	13	*N*_*s*_, Co, NW, SS, NH, Ty, RP, HT, He, FD, ND, Pa, Btr
	II	13	*N*_*s*_, Co, NW, SS, NH, Ty, RP, HT, He, ND, Pa, FD, Btl
	III	11	*N*_*s*_, RP, NW, Ty, NH, SS, Pa, He, HT, ND, FD
	IV	13	*N*_*s*_, Pa, NW, SS, NH, Ty, RP, ND, HT, He, Btl, SA, FD
	V	13	*N*_*s*_, Pa, NW, SS, Ty, NH, RP, He, HT, ND, Btr, SA, FD

BMSF	I	8	*N*_*s*_, RP, NW, PDR, HT, Bar, SS, Ta2
	II	6	*N*_*s*_, Btol, NW, HT, Bar, Ta2
	III	8	*N*_*s*_, Pa, NW, HT, Bar, Ta2, Bal, Lpd
	IV	7	*N*_*s*_, Lpd, NW, Pa, PDR, Ta2, HT
	V	8	*N*_*s*_, Btr, NW, HT, Bar, Ta2, PDR, SA

**Table 4:** Classification results with different classification models.

Feature selection methods	Naïve Bayes (%)	BPNN (%)	SVC (%)	Average (%)
All features	61.35 ± 26.82	91.46 ± 1.22	97.10 ± 0.43	83.30
SVM-RFE	30.78 ± 12.94	91.38 ± 0.83	93.29 ± 1.20	71.82
Rough set	51.30 ± 3.59	92.75 ± 0.46	93.05 ± 1.45	79.03
BMSF	70.53 ± 6.36	91.46 ± 1.45	**97.84 ± 0.57**	86.61

Average (%)	50.87	91.86	94.73	

**Table 5:** Breakdown of independent tests results of different models (%).

Classifier	FS method	Pyramidal	Motoneuron	Sensory	Tripolar	Bipolar	Multipolar	Purkinje
NB	All	30.96 ± 1.96	18.24 ± 3.12	41.62 ± 5.47	61.26 ± 7.90	94.16 ± 4.74	**98.34 ± 3.71**	96.00 ± 8.94
	SVM-RFE	29.22 ± 16.4	22.31 ± 3.26	29.80 ± 60.5	56.25 ± 9.66	92.50 ± 6.18	88.33 ± 21.73	96.0 ± 8.94
	Rough set	52.38 ± 4.48	22.32 ± 3.08	39.20 ± 3.95	**97.5 ± 2.7**	94.16 ± 6.98	85.0 ± 10.87	96.0 ± 8.94
	BMSF	77.26 ± 7.67	25.38 ± 3.73	38.93 ± 4.63	60.83 ± 4.0	90.83 ± 5.43	51.67 ± 21.57	92.0 ± 17.89

BPNN	All	99.10 ± 0.75	82.46 ± 9.78	57.84 ± 19.64	42.94 ± 11.76	0.00 ± 0.00	0.00 ± 0.00	52.00 ± 48.17
	SVM-RFE	99.12 ± 0.36	83.22 ± 18.44	45.24 ± 23.59	62.50 ± 9.64	15.84 ± 35.42	0.00 ± 0.00	80.0 ± 34.61
	Rough set	99.08 ± 0.36	78.92 ± 4.37	71.80 ± 3.09	57.06 ± 12.1	0.00 ± 0.00	0.00 ± 0.00	76.0 ± 8.94
	BMSF	98.42 ± 1.08	72.00 ± 6.01	66.16 ± 16.64	60.0 ± 18.47	14.16 ± 31.67	0.00 ± 0.00	76.0 ± 43.36

SVC	All	99.56 ± 0.18	82.46 ± 6.95	93.69 ± 5.23	87.50 ± 6.07	97.5 ± 2.28	18.33 ± 17.07	**96.0 ± 8.94**
	SVM-RFE	99.55 ± 0.13	65.38 ± 4.58	69.66 ± 15.64	69.58 ± 7.00	72.5 ± 31.26	0.00 ± 0.00	88.0 ± 17.89
	Rough set	99.52 ± 0.11	77.54 ± 5.58	54.23 ± 15.09	67.08 ± 7.71	89.17 ± 5.59	0.00 ± 0.00	92.0 ± 10.95
	BMSF	**99.63 ± 0.14**	**92.46 ± 4.9**	**95.84 ± 1.10**	83.33 ± 7.37	**99.17 ± 1.86**	1.67 ± 3.73	92.0 ± 17.89

**Table 6:** Ability to distinguish one single cell type from others and the obtained private feature subsets by BMSF-SVC.

Positive versus negative cell type	Accuracy (%)	MCC (%)	Recall (%)	Private feature subsets
{Pyramidal} versus {motoneuron, sensory, tripolar, bipolar, multipolar, Purkinje}	99.10 ± 0.12	97.05 ± 0.40	99.76 ± 0.10	*N*_*s*_, Lpd, NW, Co, Pk2, HT, Bar, Su, PDR, Ta2
				*N*_*s*_, Lpd, NW, Bal, Pk2, Vo, Bar, HT, PDR, Ta2
				*N*_*s*_, Lpd, NW, Bal, Pk2, Vo, Bar, HT, Pc, Ta2
				*N*_*s*_, Lpd, Bal, NW, HT, Pc, Vo, PDR, Ta2
				*N*_*s*_, Lpd, Co, NW, HT, PDR, Vo, Bar, Ta2

{Motoneuron} versus {sensory, tripolar, bipolar, multipolar, Purkinje}	97.26 ± 1.44	94.3 ± 3.02	94.50 ± 5.21	SS
				SS, NH, *N*_*s*_, Ta1, SA, Ta2, HT, Su, NW, Vo
				SS, NH, *N*_*s*_, HT, Vo, NW, Lpd, Dp, Su, SA
				SS, NH, *N*_*s*_, Lpd, Ta1, Vo, HT, Le, Ta2, SA
				SS, NH, *N*_*s*_, HT, SA, Ta1, Vo, ND, Le, Dp

{Sensory} versus {tripolar, bipolar, multipolar, Purkinje}	90.15 ± 1.24	80.62 ± 2.46	97.85 ± 1.38	Pa
				Pa, SS, SA, Ta1, ND, Pk2, Btr, NW, Pk, Btl
				Pa, SS, SA, Ta1, ND, Ty, Co, Di, Btr
				Pa, SS, SA, Ta1, ND, Ty, Di
				Pa, SS, SA, ND, Ta1, Ty, Btr, NW, Lpd, Pk

{Tripolar} versus {bipolar, multipolar, Purkinje}	99.16 ± 0.56	98.32 ± 1.12	99.17 ± 1.41	NW
				NW, SS, He, Pa, ND, *N*_*s*_
				NW, SS, He
				NW, SS, He
				NW, SS, He, Pa, ND

{Bipolar} versus {multipolar, Purkinje}	96.95 ± 3.07	93.86 ± 6.24	95.83 ± 2.95	*N*_*s*_
				*N*_*s*_, Vo, He, Ty, Su
				*N*_*s*_, Vo, Su, Ty, He, Ta2, NW, Btor, Pk
				*N*_*s*_, Vo, Su, NW, Ta2, Di, Pc, He, Btor
				*N*_*s*_, Vo, Su, He, Di, Ta2, Btor, Pk

{Multipolar} versus {Purkinje}	100.00 ± 0.00	100.00 ± 0.00	100.00 ± 0.00	DR
				DR
				Pa
				*N*_*s*_
				Pa

## References

[1] Bota M., Swanson L. W. (2007). The neuron classification problem. *Brain Research Reviews*.

[2] Renehan W. E., Jin Z., Zhang X., Schweitzer L. (1996). Structure and function of gustatory neurons in the nucleus of the solitary tract. II. Relationships between neuronal morphology and physiology. *The Journal of Comparative Neurology*.

[3] Badea T. C., Nathans J. (2004). Quantitative analysis of neuronal morphologies in the mouse retina visualized by a using a genetically directed reporter. *Journal of Comparative Neurology*.

[4] Kong J. H., Fish D. R., Rockhill R. L., Masland R. H. (2005). Diversity of ganglion cells in the mouse retina: unsupervised morphological classification and its limits. *Journal of Comparative Neurology*.

[5] Ristanović D., Milošević N. T., Stefanović B. D., Marić D. L., Rajković K. (2010). Morphology and classification of large neurons in the adult human dentate nucleus: a qualitative and quantitative analysis of 2D images. *Neuroscience Research*.

[6] Ascoli G. A., Donohue D. E., Halavi M. (2007). NeuroMorpho.Org: a central resource for neuronal morphologies. *The Journal of Neuroscience*.

[7] Li C., Xie X., Wu X. A universal neuronal classification and naming scheme based on the neuronal morphology.

[8] Jiang R., Liu Q., Liu S. (2011). A proposal for the morphological classification and nomenclature of neurons. *Neural Regeneration Research*.

[9] Kerschen G., Golinval J. C. (2002). Non-linear generalization of principal component analysis: from a global to a local approach. *Journal of Sound and Vibration*.

[10] Hedenfalk I., Duggan D., Chen Y. (2001). Gene-expression profiles in hereditary breast cancer. *The New England Journal of Medicine*.

[11] Iyer V. R., Eisen M. B., Ross D. T. (1999). The transcriptional program in the response of human fibroblasts to serum. *Science*.

[12] Jin X., Xu A., Bie R., Guo P. (2006). Machine learning techniques and chi-square feature selection for cancer classification using SAGE gene expression profiles. *Data Mining for Biomedical Applications*.

[13] Dash M., Liu H. (1997). Feature selection for classification. *Intelligent Data Analysis*.

[14] Kenji K., Larry A. R., Swartout W. The feature selection problem: traditional methods and a new algorithm.

[15] Golub T. R., Slonim D. K., Tamayo P. (1999). Molecular classification of cancer: class discovery and class prediction by gene expression monitoring. *Science*.

[16] Fang Z., Du R., Cui X. (2012). Uniform approximation is more appropriate for wilcoxon rank-sum test in gene set analysis. *PLoS ONE*.

[17] Zhu S., Wang D., Yu K., Li T., Gong Y. (2010). Feature selection for gene expression using model-based entropy. *IEEE/ACM Transactions on Computational Biology and Bioinformatics*.

[18] Peng H., Long F., Ding C. (2005). Feature selection based on mutual information: criteria of max-dependency, max-relevance, and min-redundancy. *IEEE Transactions on Pattern Analysis and Machine Intelligence*.

[19] Wang Y., Tetko I. V., Hall M. A. (2005). Gene selection from microarray data for cancer classification—a machine learning approach. *Computational Biology and Chemistry*.

[20] Han M., Liu X. (2012). Forward feature selection based on approximate Markov blanket. *Advances in Neural Networks—ISNN 2012*.

[21] Kittler J., Chen C. H. (1978). Feature set search algorithms. *Pattern Recognition and Signal Processing*.

[22] Pudil P., Novovičová J., Kittler J. (1994). Floating search methods in feature selection. *Pattern Recognition Letters*.

[23] Hu B. Q., Chen R., Zhang D. X., Jiang G., Pang C. Y. Ant colony optimization vs genetic algorithm to calculate gene order of gene expression level of Alzheimer's disease.

[24] Cai L. J., Jiang L. B., Yi Y. Q. (2008). Gene selection based on ACO algorithm. *Application Research of Computers*.

[25] Guyon I., Weston J., Barnhill S., Vapnik V. (2002). Gene selection for cancer classification using support vector machines. *Machine Learning*.

[26] Liu Q., Sung A. H., Chen Z. (2011). Gene selection and classification for cancer microarray data based on machine learning and similarity measures. *BMC Genomics*.

[27] Li X., Peng S., Chen J., Lü B., Zhang H., Lai M. (2012). SVM-T-RFE: a novel gene selection algorithm for identifying metastasis-related genes in colorectal cancer using gene expression profiles. *Biochemical and Biophysical Research Communications*.

[28] Kandaswamy K. K., Chou K. C., Martinetz T. (2011). AFP-Pred: a random forest approach for predicting antifreeze proteins from sequence-derived properties. *Journal of Theoretical Biology*.

[29] Ascoli G. A. (2002). *Computational Neuroanatomy: Principles and Methods*.

[30] Ascoli G. A., Krichmar J. L., Nasuto S. J., Senft S. L. (2001). Generation, description and storage of dendritic morphology data. *Philosophical Transactions of the Royal Society, Series B: Biological Sciences*.

[31] Scorcioni R., Polavaram S., Ascoli G. A. (2008). L-measure: a web-accessible tool for the analysis, comparison and search of digital reconstructions of neuronal morphologies. *Nature Protocols*.

[32] Zhang H., Wang H., Dai Z., Chen M. S., Yuan Z. (2012). Improving accuracy for cancer classification with a new algorithm for genes selection. *BMC Bioinformatics*.

[33] Pawlak Z. (1991). *Rough Set: Theoretical Aspects of Reasoning about Data*.

[34] Cao Y., Liu S., Zhang L., Qin J., Wang J., Tang K. (2006). Prediction of protein structural class with Rough Sets. *BMC Bioinformatics*.

[35] Hu Q., Yu D., Xie Z. (2006). Information-preserving hybrid data reduction based on fuzzy-rough techniques. *Pattern Recognition Letters*.

[36] Hu Q., Yu D., Xie Z., Liu J. (2006). Fuzzy probabilistic approximation spaces and their information measures. *IEEE Transactions on Fuzzy Systems*.

[37] Hu Q., Yu D. (2004). Entropies of fuzzy indiscernibility relation and its operations. *International Journal of Uncertainty, Fuzziness and Knowledge-Based Systems*.

[38] Vapnik V. N. (2000). *The Nature of Statistical Learning Theory*.

[39] Hecht-Nielsen R. Theory of the backpropagation neural network.

[40] Hall M., Frank E., Holmes G., Pfahringer B., Reutemann P., Witten I. H. (2009). The WEKA data mining software. *ACM SIGKDD Explorations Newsletter*.

[41] Mitchell T. M. (1997). *Machine Learning*.

[42] Tang Y., Zhang Y. Q., Chawla N. V., Krasser S. (2009). SVMs modeling for highly imbalanced classification. *IEEE Transactions on Systems, Man, and Cybernetics Part B: Cybernetics*.

